# High prevalence and co-occurrence of modifiable risk factors for non-communicable diseases among university students: a cross-sectional study

**DOI:** 10.3389/fpubh.2024.1484164

**Published:** 2025-01-08

**Authors:** Ipek Cicekli, Serap Gokce Eskin

**Affiliations:** ^1^Faculty of Health Sciences, Department of Nutrition and Dietetics, Acibadem University, Istanbul, Türkiye; ^2^Institute of Health Sciences, Department of Nutrition and Dietetics, Acibadem University, Istanbul, Türkiye; ^3^Faculty of Nursing, Department of Medical Nursing, Aydin Adnan Menderes University, Aydin, Türkiye; ^4^Institute of Health Sciences, Department of Medical Nursing, Aydin Adnan Menderes University, Aydin, Türkiye

**Keywords:** noncommunicable diseases, primary prevention, risk factors, students, diet, sedentary behavior

## Abstract

**Background:**

Non-communicable diseases (NCDs) are a major global concern. This study aimed to examine the prevalence and co-occurrence of lifestyle risk factors among university students.

**Methods:**

This analytical, cross-sectional study was conducted between January and April 2022. A total of 485 students were included in the study. Lifestyle risk variables for NCDs included physical inactivity, low fruit and vegetable consumption, alcohol use, tobacco smoking, being overweight/obese, and sugar sweetened beverages consumption.

**Results:**

Our results showed that the most frequent risk factor was insufficient physical activity (89.2%), followed by low vegetable (70.5%) and fruit consumption (58.9%). Overall, more than half of the students (51.2%) had four or more risk factors highlighting the urgent need for preventive interventions. The co-occurrence of four or more lifestyle risk factors was significantly greater in students at private universities (aOR: 2.01 95% CI: 1.2; 3.35), those living in student homes (aOR: 3.57 95% CI: 1.96; 6.5), and those with fast food preferences when eating outside (a0R: 2.53 95% CI: 1.62; 3.96).

**Conclusion:**

Targeted university-based interventions, such as promoting physical activity, providing affordable nutritious meals, and educating students on healthy lifestyles, are essential to reduce non-communicable disease (NCD) risk among students. Early action fosters lifelong healthy habits, supports healthy aging, and reduces healthcare costs. Future research should focus on refining these strategies to maximize their impact on university populations.

## 1 Introduction

Non-communicable diseases (NCDs) are a major cause of mortality worldwide with their prevalence rising steadily across all age groups (1). Conditions such as cardiovascular disease and diabetes are increasingly prevalent, driven by modifiable risk factors including poor diet quality, tobacco use, excessive alcohol consumption and insufficient physical activity. To combat NCD-attributable deaths, policies that focus reducing these risk factors and promoting healthy behaviors are essential (2). Behavioral risk factors for NCDs are commonly established during adolescence or early adulthood (3). Behaviors established during young adulthood can persist into later life, increasing the likelihood of developing chronic conditions such as cardiovascular diseases, diabetes, and certain cancers. Moreover, the co-occurrence of multiple risk factors can exacerbate health outcomes, leading to a greater burden of disease (4).

Among young adults, particularly university students, the prevalence of these risk factors is notably high. Studies indicate significant levels of clustering risk factors in this demographic (5). For instance, a study across 24 countries found that 15.9% of university students exhibited three or more behavioral NCD risk factors. Among these, inadequate fruit and vegetable intake was reported by 80.5% of students while 23.1% were classified as physically inactive (6). Another study investigating behavioral risk factors for cardiovascular diseases—such as being overweight, avoidance of dietary fat, low physical activity, tobacco use and excessive drinking—among university students in nine Association of Southeast Asian Nations (ASEAN) countries reported a high prevalence of these risk factors, emphasizing the need for targeted interventions by university health centers and health promotion programs (7).

University students especially vulnerable to NCD risk factors. Research conducted in Bangladesh revealed that over half of the students have obesity (50.4%), with notable prevalence of hyperglycemia (13.5%), and hypertension (12.0%). These findings underscore the critical need for targeted health interventions aimed at lifestyle modifications within this population (8). Early interventions, such as educational programs promoting physical activity and balanced nutrition, could play a pivotal role in preventing the progression of NCDs and reducing the long-term health burden (9).

The impact of NCDs is profound, affecting individuals, families, and national healthcare systems. Direct costs, including medical treatments and hospitalizations, place significant strain on health systems. In the United States and Germany, NCD-related expenditures account for 45 and 51% of healthcare costs, respectively (10). Indirect costs, such as lost productivity, transportation for healthcare access, and employer burdens due to absenteeism, further exacerbate the economic toll. Without preventive measures, these costs will continue to climb (10).

This study aims to assess the prevalence and co-occurrence of lifestyle-related risk factors for NCDs, including poor nutrition, physical inactivity, tobacco smoking, alcohol use, sugar-sweetened beverage (SSB) consumption, and overweight/obesity. The research focuses on students enrolled in the Faculty of Health Sciences at one public university and two private universities, providing insights into the health behaviors of young adults in higher education settings. By identifying these patterns, this study seeks to inform effective public health strategies and interventions that address the rising burden of NCDs.

## 2 Methods

This analytical cross-sectional study was conducted over 3 months (January–April 2022) at the Faculty of Health Sciences in two universities—one public and one private. Students were approached in their classrooms before the start of classes, and written informed consent was obtained from all participants.

A structured data collection form, developed by the researchers based on a comprehensive literature review, was used to assess sociodemographic characteristics and lifestyle risk factors for NCDs. Content validity was ensured through expert review by researchers in nutrition and public health, who provided feedback on the relevance and clarity of the questions. The form was administered to students face-to-face, with each interview lasting approximately 15 min. The assessed lifestyle risk factors for NCDs included physical inactivity, low fruit and vegetable intake, alcohol consumption, tobacco use, being overweight or obese, and SSB consumption. Researchers also collected anthropometric measurements along with self-reported data on health, household characteristics, and living conditions.

### 2.1 Population

Students in the Nutrition and Dietetics program who were aged between 18 and 25 years, present during the classroom sessions and willing to complete the data collection form were included in the study. Students absent during data collection or failing to complete the form were excluded. In this study, purposive sampling (a type of non-probability sampling) was employed to target a specific population of interest—nutrition and dietetics students—due to their relevance to the study objectives. This approach was chosen to ensure that the sample represented students with a foundational understanding of health and nutrition, making them a critical population for assessing the prevalence and co-occurrence of non-communicable disease (NCD) risk factors. To ensure an adequate sample size, a power analysis was conducted. Based on an 80% power, 0.05 margin of error, medium effect size, and an anticipated 10% data loss, it was determined that at least 320 students were required to achieve statistical significance. Our goal was to include the entire population of nutrition and dietetics students while maintaining a robust sample size. Ultimately, a total of 485 students participated in the survey, with a response rate of 93%. The study included undergraduate students aged 18–25 years who provided informed consent. Measures were taken to reduce response bias, including anonymizing responses and emphasizing the voluntary nature of participation. While purposive sampling does not allow for generalization to all university students, it was appropriate for this study because the target population had specific characteristics critical to the research question. This method allowed us to focus on a group particularly relevant to the investigation of NCDs risk factors. Additionally, this sampling strategy was supplemented by a large sample size, which enhances the reliability of the findings within this population.

### 2.2 Questionnaire

Height (cm) and weight (kg) data were collected on the basis of self-reports, and BMI was calculated with the formula weight/height^2^ to determine whether the participants were normal weight (≤ 24.9 kg/m^2^), overweight (25.0–29.9 kg/m^2^) or obese (≤ 30 kg/m^2^) according to the World Health Organization (WHO) (11). Being overweight or obese was also considered a risk factor. The physical activity level (frequency of moderate-or high-intensity physical activity lasting at least 30 min without interruption) was measured daily, 5–6 days a week, 2–4 days a week and ≤ 1 day a week, and the activity levels were evaluated. A physical activity level of <150 min/week (12) was considered an insufficient level of physical activity and a risk factor. Smoking status was classified as active smoker, previous smoker but later quit, or nonsmoker. Active smoking is an accepted risk factor. Alcohol consumption was assessed by asking, “Have you drank alcohol at least once in the last month?” to assess current alcohol consumption. A positive answer to this question was considered to be the use of alcohol. The frequency of alcohol use was assessed by asking about the “frequency of consuming alcoholic beverages in the amount of one glass/glass of wine” and classifying it as almost daily, 4–6 days a week, 1–3 days a week, a few days a month, less, or not at all. No safe limit exists for alcohol consumption (13, 14); therefore, it was accepted as a risk factor.

The WHO recommends consuming more than 400 g of fruits and vegetables daily to improve overall health and reduce the risk of certain NCDs (15). In this study Food Frequency Questionnaire (FFQ) was used to assess fruit and vegetables intake. Low vegetable consumption was defined as consuming four or fewer portions per day (approximately 80 g per portion), while low fruit consumption was defined similarly. For sugar-sweetened beverage (SSB) consumption, individuals who consumed SSBs at least once per week were classified as being at risk, while those who never or consumed on occasion SSBs (a few days per month) were not. These thresholds were chosen to reflect dietary behaviors associated with increased NCD risk.

### 2.3 Statistics

Data were analyzed using SPSS version 26 (16). The prevalence and 95% confidence intervals (CIs) were calculated to identify the risk factors for NCDs according to sociodemographic characteristics. Differences between the measurement variables were examined using *t*-tests. Count-type variables were compared according to sociodemographic characteristics using chi-square tests.

A total of 128 potential combinations of the seven risk factors were initially analyzed (all possible combinations of the seven variables = 2^^7^). However, certain combination groups were excluded due to the absence of participants. Finally, 77 combinations were included in the analysis. Risk factors associated with the same period were considered as co-occurring. Having four or more risk factors was considered the threshold for identifying co-occurrence, and after this threshold, the rates suddenly decreased.

A multivariate logistic regression model was built to examine the odds ratios (ORs) and 95% CIs for the associations between the determined variables (sex, age, and education level) and four or more risk factors. For all analyses, statistical significance was set at *p* < 0.05.

## 3 Results

In total, 485 (86.6% women) students, comprising 265 (54.6%) from state universities and 220 (45.4%) from private universities, were included in this study, with a mean age of 21.21 ± 3.10 years. Overall, 23.5% were in their first year of university, 30.5% in their second year, 24.3% in their third year, and 21.8% in their fourth year. A total of 34.4% of the students lived in student houses during their university education, and 29.5% lived with their families.

Table 1 shows the prevalence and 95% CIs of the risk factors determined for the students. Overall, the most frequent risk factors were insufficient physical activity (89.2%), low fruit consumption (58.9%), low vegetable consumption (70.5%), alcohol consumption (44.5%), SSB consumption (41.1%), tobacco smoking (25.2%), and being overweight or obese (14.9%). Students living in their homes had a higher prevalence of tobacco (34.5%) and alcohol consumption (58.2%). Whereas, the prevalence of low vegetable (76.0%) and fruit (67.3%) content was higher among students living in dorms. Consumption of SSB was more prevalent among grade 1 students.

**Table 1:** Prevalence of risk factors for NCDs.

	Insufficient physical activity % (95% CI)	Tobacco smoking % (95% CI)	Alcohol consumption % (95% CI)	Low vegetable consumption % (95% CI)	Low fruit consumption % (95% CI)	Overweight or obesity % (95% CI)	SSBs consumption % (95% CI)
Total	89.2 (86.1; 91.9)	25.2 (21.6; 29.3)	44.5 (40.6; 49.1)	70.5 (66.3; 74.7)	58.9 (54.2; 63.7)	14.9 (11.8; 18.3)	41.1 (36.8; 45.5)
Gender							
Female	91.9 (88.8; 94.3)	24.8 (20.7; 29.2)	43.3 (38.5; 48.2)	70.0 (65.3; 74.4)	58.7 (53.8; 63.5)	12.2 (9.2; 15.7)	39.0 (34.1; 44.0)
Male	73.0 (60.3; 83.4)	28.1 (17.6; 40.8)	53.1 (40.2; 65.7)	74.2 (61.5; 84.5)	59.7 (46.4; 71.9)	32.8 (21.6; 45.7)	55.7 (42.4; 68.5)
Age							
18–20 years	90.2 (85.5; 93.7)	22.6 (17.3; 28.6)	43.8 (37.2; 50.5)	76.6 (70.4; 82.0)	61.3 (54.5; 67.7)	13.8 (9.6; 19.1)	50.0 (43.0; 57.0)
≥ 21 years	88.4 (83.8; 92.0)	27.4 (22.1; 33.3)	45.2 (39.0; 51.5)	65.2 (59.0; 71.1)	56.7 (50.4; 62.9)	15.9 (11.7; 20.9)	33.6 (27.7; 39.9)
Grade							
Grade 1	90.4 (83.4; 95.1)	21.1 (14.0; 29.7)	43.0 (33.7; 52.6)	69.6 (60.2; 78.0)	60.7 (51.0; 69.8)	14.2 (8.3; 22.0)	51.8 (42.1; 61.3)
Grade 2	89.7 (83.5; 94.1)	25.0 (18.3; 32.8)	40.5 (32.6; 48.9)	76.6 (68.8; 83.2)	57.6 (49.1; 65.8)	17.1 (11.4; 24.2)	41.7 (33.0; 50.8)
Grade 3	84.7 (77.0; 90.7)	24.6 (17.1; 33.4)	46.6 (37.4; 56.0)	76.9 (68.2; 84.2)	63.2 (53.8; 72.0)	13.6 (8.0; 21.1)	41.5 (32.5; 51.0)
Grade 4	92.4 (85.5; 96.7)	30.5 (21.9; 40.2)	49.5 (39.6; 59.5)	55.4 (45.2; 65.3)	53.5 (43.3; 63.5)	14.3 (8.2; 22.5)	28.0 (19.5; 37.9)
University							
Public	87.5 (83.0; 91.3)	22.3 (17.4; 27.8)	42.3 (36.2; 48.5)	69.9 (63.9; 75.5)	62.7 (56.5; 68.7)	16.7 (12.4; 21.7)	37.3 (31.4; 43.4)
Private	91.2 (86.7; 94.6)	28.6 (22.8; 35.1)	47.3 (40.5; 54.1)	71.2 (64.7; 77.1)	54.3 (47.5; 61.1)	12.8 (8.7; 18.0)	46.4 (39.2; 53.7)
Mother’s education level							
Primary or secondary school	89.7 (84.3; 93.7)	22.3 (16.5; 29.0)	34.8 (27.9; 42.1)	70.2 (62.9; 76.8)	60.1 (52.5; 67.4)	14.7 (9.9; 20.6)	36.7 (29.6; 44.3)
High school or university	89.2 (85.0; 92.5)	27.2 (22.2; 32.6)	50.7 (44.8; 56.5)	71.1 (65.5; 76.2)	58.4 (52.5; 64.1)	15.3 (11.3; 19.9)	44.0 (38.1; 50.1)
Father’s education level							
Primary or secondary school	90.8 (84.9; 95.0)	23.6 (16.9; 31.4)	38.9 (30.9; 47.4)	66.4 (58.0; 74.2)	58.6 (49.9; 66.8)	16.0 (10.4; 23.0)	37.5 (29.4; 46.2)
High school or university	88.1 (84.1; 91.4)	26.0 (21.3; 31.1)	46.8 (41.3; 52.4)	73.4 (68.2; 78.1)	59.3 (53.7; 64.7)	14.5 (10.9; 18.8)	43.0 (37.4; 48.8)
Residence during education							
Living with family	89.4 (83.1; 93.9)	18.2 (12.2; 25.5)	34.3 (26.5; 42.7)	64.5 (56.0; 72.4)	45.4 (37.0; 54.0)	14.8 (9.4; 21.7)	32.8 (24.9; 41.6)
Student house	87.9 (81.9; 92.4)	34.5 (27.3; 42.3)	58.2 (50.3; 65.8)	69.8 (62.1; 76.7)	61.5 (53.5; 69.0)	18.2 (12.6; 24.9)	39.9 (32.2; 48.0)
Dorm	90.3 (84.9; 94.2)	21.6 (15.8; 28.4)	40.3 (33.0; 48.0)	76.0 (68.9; 82.2)	67.3 (59.7; 74.2)	12.1 (7.6; 17.9)	48.5 (40.7; 56.3)

**Figure 1:**
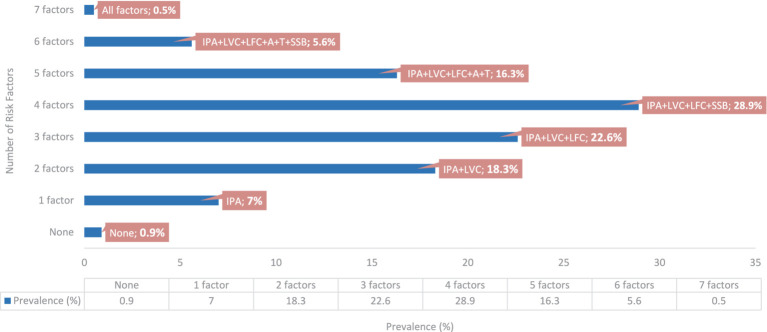
Prevalence of risk factors co-occurrence and most common combinations. IPA, insufficient physical activity; LVC, low vegetable consumption; LVF, low fruit consumption; A, alcohol consumption; T, tobacco smoking; SSB, sugar-swetened beverages consumption.

Table 2 shows the prevalence of co-occurrence for all combinations of the seven risk factors. Among the combinations, four risk factors (28.9%) had the highest prevalence, followed by three (22.6%) and two risk factors (18.3%). The highest prevalence (9.9%) observed for the combinations of risk factors was the co-occurrence of insufficient physical activity, low vegetable and fruit consumption, and SSB consumption (Figure 1). The observed prevalence in individuals with no risk factors was 0.9%, whereas that in individuals with all risk factors was 0.5%. When risk factors were examined alone, the highest prevalence was observed for insufficient physical activity (89.2, 95% CI: 86.1; 91.9), followed by low vegetable consumption (70.5, 95% CI: 66.3, 74.7), low fruit consumption (58.9, 95% CI: 54.2; 63.7), alcohol consumption (44.5, 95% CI: 40.6; 49.1), SSB consumption (41.1, 95% CI: 36.8; 45.5), tobacco smoking (25.2, 95% CI: 21.6; 29.3), and being overweight/obese (14.9, 95 %CI: 11.8; 18.3).

**Table 2:** Prevalence and co-occurrence patterns of risk factors for NCDs.

	Insufficient physical activity	Tobacco smoking	Alcohol consumption	Low vegetable consumption	Low fruit consumption	Overweight or obesity	SSB consumption	Observed prevalence (%)
7 (0.5%)	+	+	+	+	+	+	+	0.5
6 (5.6%)	+	+	+	+	+	+	−	0.2
	**+**	**+**	**+**	**+**	**+**	**−**	**+**	**3.9**
	+	+	+	−	+	+	+	0.2
	+	+	−	+	+	+	+	0.5
	+	−	+	+	+	+	+	0.9
5 (16.3%)	**+**	**+**	**+**	**+**	**+**	**−**	**−**	**5.1**
	+	−	−	+	+	+	+	0.5
	+	+	+	−	−	+	+	0.2
	−	+	+	+	+	+	−	0.2
	+	−	+	+	+	+	−	1.2
	+	−	+	−	+	+	+	0.2
	+	+	−	+	−	+	+	0.7
	+	+	+	−	+	−	+	0.2
	−	+	+	+	+	−	+	0.9
	+	−	+	+	+	−	+	4.8
	+	+	−	+	+	−	+	0.9
4 (28.9%)	+	+	+	+	−	−	−	1.8
	+	+	+	−	+	−	−	0.7
	+	+	+	−	−	−	+	0.9
	+	+	−	+	+	−	−	0.7
	+	+	−	+	−	+	−	0.5
	+	+	−	−	+	+	−	0.2
	+	+	−	−	+	−	+	0.5
	+	−	+	+	+	−	−	6.2
	+	−	+	+	−	+	−	0.9
	+	−	+	+	−	−	+	2.5
	+	−	+	−	+	+	−	0.5
	+	−	+	−	+	−	+	0.7
	+	−	+	−	−	+	+	0.5
	+	−	−	+	+	+	−	0.9
	**+**	**−**	**−**	**+**	**+**	**−**	**+**	**9.9**
	+	−	−	+	−	+	+	0.2
	+	−	−	−	+	+	+	0.2
	−	+	+	+	−	+	−	0.2
	−	+	+	−	+	+	−	0.2
	−	+	+	−	−	+	+	0.5
	−	+	−	+	+	+	−	0.2
	−	−	−	+	+	+	+	0.2
	−	−	+	+	+	−	+	0.2
3 (22.6%)	−	−	−	+	+	−	+	0.2
	−	−	+	−	+	+	−	0.2
	−	−	+	+	−	−	+	0.2
	−	+	+	−	−	−	+	0.2
	−	+	+	−	−	+	−	0.2
	−	+	+	+	−	−	−	0.2
	+	−	−	−	−	+	+	0.7
	+	−	−	−	+	−	+	0.7
	+	−	−	+	−	−	+	2.3
	+	−	−	+	−	+	−	0.9
	**+**	**−**	**−**	**+**	**+**	**−**	**−**	**9.7**
	+	−	+	−	−	−	+	0.9
	+	−	+	−	+	−	−	1.6
	+	−	+	+	−	−	−	2.8
	+	+	−	−	−	−	+	0.2
	+	+	+	−	−	−	−	0.7
	+	+	−	−	+	−	−	0.7
	+	+	−	+	−	−	−	0.2
2 (18.3%)	−	−	−	−	−	+	+	0.5
	+	+	−	−	−	−	−	0.7
	−	+	+	−	−	−	−	0.2
	−	−	+	+	−	−	−	0.5
	−	−	−	+	+	−	−	1.2
	−	−	−	−	+	+	−	0.2
	+	−	−	−	−	−	+	2.5
	−	+	−	−	−	−	+	0.2
	+	−	+	−	−	−	−	1.4
	−	+	−	+	−	−	−	0.2
	**+**	**−**	**−**	**+**	**−**	**−**	**−**	**5.5**
	+	−	−	−	+	−	−	3.5
	+	−	−	−	−	+	−	1.4
	−	−	+	−	−	+	−	0.2
	−	+	−	−	+	−	−	0.2
1 (7.0%)	−	−	−	+	−	−	−	0.9
	−	−	+	−	−	−	−	0.2
	**+**	**−**	**−**	**−**	**−**	**−**	**−**	**6.2**
0 (0.9%)	−	−	−	−	−	−	−	0.9

The symbol ‘+’ represents the presence of the factor, and ‘–’ represents the absence of the factor. The bold values represent *p* < 0.005 and indicate statistical significance.

Overall, 51.2% (95% CI: 46.5; 56.0) of the students had four or more risk factors. Students with four or more risk factors were more likely to live in student houses (39.1%) and preferred fast food when eating out (59.5%). Moreover, among students with four or more risk factors, the prevalence of students with five or more hours of daily screen time was higher (60.1%) than that among those with fewer risk factors. The average daily water intake was higher (26.22 ± 11.14 mL) in students with three or more risk factors (Table 3).

**Table 3:** Comparison of sociodemographic variables by the presence of four or more risk factors for NCDs.

		Three or less risk factors	Four or more risk factors	*p*
Overall		48.8% (44.0; 53.5)	51.2% (46.5; 56.0)	
Age (*n* = 443)	18–20 years	90 (41.7%)	114 (50.2%)	0.071
	≥21 years	126 (58.3%)	113 (49.8%)	
Gender (*n* = 443)	Female	192 (88.9%)	193 (85.0%)	0.228
	Male	24 (11.1%)	34 (15.0%)	
University (*n* = 443)	Public	131 (60.6%)	122 (53.7%)	0.142
	Private	85 (39.4%)	105 (46.3%)	
Grade (*n* = 443)	Grade 1	50 (23.1%)	59 (26.0%)	0.424
	Grade 2	57 (26.4%)	64 (28.2%)	
	Grade 3	55 (25.5%)	62 (27.3%)	
	Grade 4	54 (25.0%)	42 (18.5%)	
Residence (*n* = 432)	Living with family	74 (34.9%)	52 (23.6%)	**0.026**
	Student house	64 (30.2%)	86 (39.1%)	
	Dorm	74 (34.9%)	82 (37.3%)	
Frequency of eating outside (*n* = 433)	Less than once a week	83 (39.2%)	45 (20.4%)	**<0.001**
	1–3 times per week	87 (41%)	102 (46.2%)	
	Four or more times a week	42 (19.8%)	74 (33.5%)	
Preference of eating outside (*n* = 431)	In dining hall or one-pot meals	103 (61.7%)	64 (38.3%)	**<0.001**
	Fast food	107 (40.5%)	157 (59.5%)	
Daily screen time (*n* = 434)	Less than 5 h	107 (50.7%)	89 (39.9%)	**0.020**
	5–9 h	97 (46.0%)	116 (52.0%)	
	10 h or more	7 (3.3%)	18 (8.0%)	
		Mean ± SD	Mean ± SD	
		4.77 ± 2.17	5.36 ± 2.38	**0.007**
Daily water intake (ml/kg) (*n* = 420)		26.22 ± 11.14	23.96 ± 11.08	**0.038**

SD, Standard deviation. h, hours. The bold values represent *p* < 0.005 and indicate statistical significance.

The results of the multivariate logistic regression analysis for the co-occurrence of four or more risk factors for NCDs are shown in Table 4. The co-occurrence of four or more lifestyle risk factors was significantly higher among students at private universities (aOR: 2.01 95% CI: 1.2; 3.35), those living in student homes (aOR: 3.57 95% CI: 1.96; 6.5), and those with fast food preferences when eating outside (a0R: 2.53 95% CI: 1.62; 3.96). Conversely, a reverse association was observed between daily water intake (a0R: 0.98 95% CI: 0.96; 0.998) and four or more risk factors.

**Table 4:** Multivariate logistic regression analysis of sociodemographic characteristics associated with the co-occurrence of four or more risk factors (*n* = 407; *R*^2^ = 0.164).

	aOR (95% CI)	*p*
University^Public^		
Private	2.01 (1.2; 3.35)	**0.008**
Grade^Grade 1^		0.505
Grade 2	0.98 (0.53; 1.79)	0.941
Grade 3	1.00 (0.53; 1.89)	1.00
Grade 4	0.62 (0.28; 1.35)	0.226
Age^18–20 years^		
>21 years	0.98 (0.59; 1.65)	0.946
Gender^Female^		
Male	1.67 (0.88; 3.19)	0.117
Mother’s education level^high school or university^		
Primary or secondary school	0.73 (0.47; 1.14)	0.169
Residence^Living with family^		**<0.001**
Student house	3.57 (1.96; 6.5)	**<0.001**
Dorm	2.47 (1.34; 4.56)	**0.004**
Daily screen time^Less than 5 h^		0.459
5–9 h	1.25 (0.80; 1.95)	0.329
10 h or more	1.67 (0.61; 4.62)	0.322
Daily water intake (ml/kg)	0.98 (0.96; 0.998)	**0.031**
Preferences of eating outside ^In dining hall or one-pot meals^		
Fast food	2.53 (1.62; 3.96)	**<0.001**

OR, odds ratio; 95% CI, confidence interval 95%. References are shown as superscripts. The bold values represent *p* < 0.005 and indicate statistical significance.

## 4 Discussion

This study evaluated the prevalence and co-occurrence of the modifiable lifestyle risk factors for NCDs among university students, considering their sociodemographic characteristics. A significant prevalence of co-occurring risk factors was observed, with over half of the participants presenting four or more risk factors. This underscores the compounded vulnerability of university students to lifestyle-related NCDs.

### 4.1 Insufficient physical activity

The prevalence of insufficient physical activity was alarmingly high at 89.2%, consistent with global trends among university students. This rate is concerning, as sedentary behavior is a well-established risk factor for non-communicable diseases (NCDs), including cardiovascular disease, diabetes, and certain cancers (17). Approximately 81% of adolescents worldwide fail to meet physical activity guidelines and more girls inactive than boys in most countries, reflecting a pervasive issue (18). In 2012, a related study investigating the impact of physical inactivity on major NCDs globally revealed striking finding: The research projected that eliminating physical inactivity could result in a median increase in life expectancy of 0.68 years worldwide, 0.63 years in Europe, and 1.06 years in Turkey (17). Academic life, characterized by prolonged sedentary behavior, screen time, and limited access to recreational facilities, likely contributes to these patterns particularly for university students (19). These findings emphasizes the critical need for specific solutions to promote physical activity among university students. Policymakers and university administrators must prioritize accessible fitness facilities, structured physical activity programs, and campus-wide campaigns to encourage active lifestyles.

### 4.2 Low fruit and vegetable consumption

In this study, roughly 7 in 10 students had low vegetable consumption, and 6 in 10 students had low fruit consumption. A study assessing university students from 26 countries across America, Asia, and Africa and reported a great prevalence (82.8%) of inadequate fruit and vegetable consumption, underscoring the need for improved intake among university students globally which aligns with these findings (20). University students were more likely to have a low frequency of fruit and vegetable consumption suggesting that research conducted among young adults, particularly university students, has reported a high prevalence of insufficient fruit and vegetable consumption. For instance, rates were 85.2% in Brazil (21), 95% in Germany (22), 73.6% in Saudi Arabia (23), and 70% in the UK (24). Financial constraints, time limitations, and easy availability of processed foods often dictate dietary habits of university students (5). Adequate fruit and vegetable intake is crucial for providing necessary nutrients and dietary fiber, vitamin and minerals which protect against NCDs (25). Policies to subsidize healthy food options, increase access to fresh produce on campuses, and integrate nutrition education into health programs are essential.

### 4.3 SSB consumption

The excessive intake of SSBs by university students is a major public health concern. Since added sugars in beverages raise blood glucose and insulin levels, which may lead to an increased risk of type 2 diabetes (26). In this study, over 2 in 5 students reported frequent SSB intake. Younger populations are particularly susceptible due to the affordability, availability, and aggressive marketing of sugary drinks (27). In the United States, the National Longitudinal Study of Adolescents and Adults Health reported a high prevalence of SSB consumption, with 87.3% of participants having consumed SSBs in the previous week and 47.8% consuming eight or more such beverages. Additionally, young adults who consume sugar-or artificially sweetened beverages tend to have a higher cumulative disease burden.

From another perspective, there has been growing interest in encouraging water consumption as a strategy to reduce SSB intake, with the expectation that increasing water consumption can decrease SSB consumption by replacing it. Notably, students with four or more risk factors were found to have lower water intake, highlighting an inverse association. This suggests that encouraging higher water consumption could be an effective approach to reducing SSB intake. Supporting this, a systematic review concluded that promoting water intake can significantly decrease SSB consumption (28). These findings suggest that public health messages for young adults should include warnings about both sugar-sweetened and artificially sweetened beverages (29). To mitigate these risks, public health measures such as taxation on sugary drinks, educational campaigns, increasing the availability of healthier beverage options like water should be prioritized.

### 4.4 Alcohol consumption and tobacco use

Tobacco smoking and alcohol consumption are prevalent risk behaviors among university students, with this study reporting rates of 25.2% for smoking and 44.5% for alcohol use. Similarly, a study conducted among university students in China reported that 29.8% of students smoked or used e-cigarettes (30). These behaviors are often influenced by peer pressure, stress, and normalization within student cultures. University students aged between 18 and 25 are at greater risk of initiating tobacco smoking due to the transition point from high school to college (31) Universities, therefore, ought to allocate resources toward the prevention and treatment of students at risk for alcohol, smoking and drug use disorders to reduce the effects on their academic performance and mental health throughout their university experience (31).

### 4.5 Co-occurrence of risk factors

A significant prevalence of risk factor co-occurrence was found in more than half of the research population (51.2%) which had four or more risk factors. Moreover, a reverse association was observed between daily water intake and four or more risk factors. Previous studies have shown that risky behaviors commonly co-occur, with 52% in the USA (32), 55% in the Netherlands (33), 59% in Brazil (17), and 68% in England (34). Accordingly, four lifestyle risk variables (inadequate fruit and vegetable intake, smoking, alcohol consumption, and poor physical activity) were studied, focusing on the co-occurrence and clustering profiles of cardiovascular lifestyle risk factors among adults in West Africa. The prevalence of two or more cardiovascular lifestyle risk factors co-occurring was 46.4% (35).

This study revealed that the most common combination of risk factors was the co-occurrence of insufficient physical activity, low vegetable and fruit consumption, and SSB consumption, indicating a group with an increased risk of NCDs. Similarly, a systematic review revealed an especially high prevalence of insufficient physical activity and low fruit and vegetable intake (36). These findings are consistent with data from other countries, including the United States (37), where low fruit and vegetable intake and physical inactivity were the most common co-occurring behaviors. Most studies have focused on adult populations at the center, with few studies considering younger adults or students. Notably, the primary limitation of the studies with co-occurrence was the fluctuating cutoff values for risk attitudes. These limitations make it harder to compare studies and are likely to contribute to the observed variability in most data.

In this study, students with four or more risk factors are less likely to live with their families compared to those with fewer risk factors. This finding aligns with literature suggesting that students living away from family often experience fewer home-cooked meals due to factors like limited cooking facilities and time constraints, thus increasing their susceptibility to poor dietary patterns (38).

This study revealed an inverse relationship between daily water intake and the presence of four or more risk factors. Although no clustered studies exist on the co-occurrence of risk factors for NCDs and daily water consumption, one prospective study reported that higher water intake was associated with a lower risk of mortality (39). Similarly, a systematic review of prospective cohort studies demonstrated that higher total water consumption was associated with a decreased risk of cardiovascular diseases mortality (40). Because proper hydration and water consumption are required for important physiological and metabolic functions, understanding the relationship between water consumption and NCD risk factors is critical for policymakers.

Research on this topic is exceptionally heterogeneous, with different approaches for defining and assessing risk factors, and no consensus on which risk factors typically occur together (41). Therefore, studies that cluster and comprehend the different risk factors for NCDs and include different age groups, especially among university students, are needed.

### 4.6 Strengths and limitations

This study’s strengths include its comprehensive analysis of multiple NCD risk factors and their co-occurrence, addressing a critical gap in the literature where studies on the co-occurrence of risk factors among university students are notably scarce. By examining variables like residence, eating habits, and screen time, it provides valuable insights into clustering behaviors. The use of robust statistical methods enhances reliability, while the focus on an underrepresented population contributes to global NCD prevention efforts.

This study had several limitations. First, its cross-sectional design limits the ability to establish causal relationships. The dependence on self-reported data may lead to recall and social desirability biases, as participants could understate undesirable actions or overstate behaviors deemed socially acceptable. To mitigate this, anonymity was ensured during data collection. Nonetheless, the absence of a fully standardized and formally validated questionnaire may affect the accuracy and reliability of self-reported behaviors. Furthermore, the purposive sampling method limits the generalizability of the findings to university students from institutions with different cultural, geographical, and academic characteristics. The predominance of female participants and the inclusion of only two universities, one private and one public, may also have introduced selection bias. Future research using randomized sampling across diverse settings and validated instruments is needed to confirm and expand upon these findings.

## 5 Conclusion

Our findings highlight the urgent need for university-based interventions targeting physical activity and dietary habits to reduce the risk of non-communicable diseases (NCDs) among students. Programs that promote structured physical activity, collaborative efforts between university administrations and public health bodies to implement wellness programs, provide healthier, nutritious, affordable dining options on campus and educate students on healthy lifestyle choices could play a pivotal role in mitigating these risks. The identification of multiple risk factors in many students underscores the necessity of early action. Intervening during university years not only fosters healthier habits that persist into adulthood but also contributes to healthy aging, reducing the burden of NCDs later in life. Additionally, such proactive measures can lower both the direct costs (e.g., treatment expenses) and indirect costs (e.g., lost productivity) for healthcare systems. Future research should explore diverse clusters of risk factors and comprehensive data to refine these strategies and optimize their impact on university populations.

## Data Availability

The data that support the findings of this study are available from the authors, upon reasonable request.
