# Supplementary material for: High prevalence and co-occurrence of modifiable risk factors for non-communicable diseases among university students: a cross-sectional study
Source: Front Public Health. 2025 Jan 8;12:1484164. doi: 10.3389/fpubh.2024.1484164 (PMC11753351; doi:10.3389/fpubh.2024.1484164)
Supplement: Supplementary file 1 [file Table_1.DOC]

**Questionnaire**

**(Form 9)**

**No:**

1. Age: ……. years
2. Gender: 1. Female 2. Male 3. I don't want to specify
3. Height: ……. cm
4. Weight: …….. kg
5. University: 1. Public 2. Private
6. Grade:
7. Grade 1
8. Grade 2
9. Grade 3
10. Grade 4
11. How many hours a day do you spend in front of a screen (phone, computer, TV, etc.) ?……..hours/day
12. Family education levels:

**Mother:** **Father:**

İlliterate İlliterate

Literate Literate

Primary school  Primary school

Secondary school  Secondary school

High school  High school

University University

1. Which one defines smoking status in your home?

 No smoking anywhere at home.

 Smoked only in a certain room (kitchen, living room, etc.) at home, per day.... number, …days per week

 Can be smoked anywhere at home, per day.... number, …days per week

1. Transportation to university:

By foot School service By car/bus By bicycle

1. Where you stayed during the education period?

Family house Student house Public dormitory Private dormitory

1. Which of the following options describes the frequency with which you engage in moderate or high-intensity physical exercise that lasts at least 30 minutes without interruption?

1. Every day, 2. 5-6 days per week, 3. 2-4 days per week, 4. 1 day or less per week

1. Do you smoke?

1. No 2. Yes ………. Number/day

1. Which of the following statements about smoking best describes you?

1. I smoked at least one cigarette every day in the last week.

2. I smoked occasionally in the last week

3. I smoked before and I quit.

4. I have never smoked in my life.

1. Have you drank alcohol at least once in the last month?

1. No 2. Yes

1. Which of the following options describes the frequency with which you drink alcoholic beverages, equal to one glass of beer/one glass of wine?
2. Almost every day
3. 4-6 days a week
4. 1-3 days a week
5. A few days a month
6. Less or none
7. Which of the following options describes the frequency and quantity you consume sugar-sweetened beverages? (One glass should be considered as 200 ml)
8. Almost every day ……………ml or ……..glass
9. 4-6 days a week …………….ml or …………glass
10. 1-3 days a week ……………..ml or …………glass
11. A few days a month ……………ml or …………glass
12. Less or none …………….ml or …………glass
13. How many glasses of water do you drink daily? (One glass should be considered as 200 ml) ……………………..glasses
14. How many days per week do you eat meals outside your home?
15. Almost every day
16. 4-6 days a week
17. 1-3 days a week
18. A few days a month
19. Less or none
20. Which dishes do you usually prefer when you eat outside?
21. Table d'hôte (in university’s dining hall)
22. Fast food (hamburger, pizza etc.)
23. One-pot meals (finedining etc)
24. Others …………………………………………

**Food Frequency Questionnaire**

| **Food groups** | **Every day** | **5-6 days a week** | **3-4 days a week** | **1-2 days a week** | **2 per month** | **1 per month** | **Never** | **Measure (serving size)** | **Quantity (g/ml)** |
| --- | --- | --- | --- | --- | --- | --- | --- | --- | --- |
| Dairy products |  |  |  |  |  |  |  |  |  |
| Eggs |  |  |  |  |  |  |  |  |  |
| Meat-cheese |  |  |  |  |  |  |  |  |  |
| Legumes |  |  |  |  |  |  |  |  |  |
| Vegetables |  |  |  |  |  |  |  |  |  |
| Fruits |  |  |  |  |  |  |  |  |  |
| Sweets |  |  |  |  |  |  |  |  |  |
| Packaged foods, chips, other snacks |  |  |  |  |  |  |  |  |  |
